# Red blood cells from endothelial nitric oxide synthase-deficient mice induce vascular dysfunction involving oxidative stress and endothelial arginase I

**DOI:** 10.1016/j.redox.2023.102612

**Published:** 2023-01-13

**Authors:** Zhengbing Zhuge, Sarah McCann Haworth, Carina Nihlén, Lucas Rannier R.A. Carvalho, Sophia K. Heuser, Andrei L. Kleschyov, Josefine Nasiell, Miriam M. Cortese-Krott, Eddie Weitzberg, Jon O. Lundberg, Mattias Carlström

**Affiliations:** a Department of Physiology and Pharmacology, Karolinska Institutet, Stockholm, Sweden; b Myocardial Infarction Research Laboratory, Division of Cardiology, Pulmonology and Vascular Medicine, Medical Faculty, Heinrich-Heine-University, Düsseldorf, Germany; c Department of Clinical Sciences, Karolinska Institutet, Stockholm, Sweden; d Department of Obstetrics and Gynecology, Danderyd Hospital, Stockholm, Sweden; e Department of Perioperative Medicine and Intensive Care, Karolinska University Hospital, Stockholm, Sweden

**Keywords:** Red blood cells, eNOS, Nitric oxide, Arginase, Oxidative stress

## Abstract

**Background & aims:**

Nitric oxide bioactivity (NO) from endothelial NO synthase (eNOS) importantly contributes to the maintenance of vascular homeostasis, and reduced eNOS activity has been associated with cardiovascular disease. Emerging evidence suggests interaction(s) between red blood cells (RBCs) and the endothelium in vascular control; however, the specific role of RBC eNOS is less clear. We aimed to investigate the hypothesis that a lack of RBC eNOS induces endothelial dysfunction.

**Methods & Results:**

RBCs from global eNOS knockout (KO) and wildtype (WT) mice were co-incubated *ex vivo* overnight with healthy mouse aortic rings, followed by functional and mechanistic analyses of endothelium-dependent and independent relaxations. RBCs from eNOS KO mice induced endothelial dysfunction and vascular oxidative stress, whereas WT RBC did not. No differences were observed for endothelium-independent relaxations. This eNOS KO RBC-induced endothelial dysfunctional phenotype was prevented by concomitant co-incubation with reactive oxygen species scavenger (TEMPOL), arginase inhibitor (nor-NOHA), NO donor (detaNONOate) and NADPH oxidase 4 (NOX4) inhibitor. Moreover, vessels from endothelial cell-specific arginase 1 KO mice were resistant to eNOS KO-RBC-induced endothelial dysfunction. Finally, in mice aortae co-incubated with RBCs from women with preeclampsia, we observed a significant reduction in endothelial function compared to when using RBCs from healthy pregnant women or from women with uncomplicated gestational hypertension.

**Conclusions:**

RBCs from mice lacking eNOS, and patients with preeclampsia, induce endothelial dysfunction in adjacent blood vessels. Thus, RBC-derived NO bioactivity acts to prevent induction of vascular oxidative stress occurring via RBC NOX4-derived ROS in a vascular arginase-dependent manner. Our data highlight the intrinsic protective role of RBC-derived NO bioactivity in preventing the damaging potential of RBCs. This provides novel insight into the functional relationship between RBCs and the vasculature in health and cardiovascular disease, including preeclampsia.

## Highlights

• Using a novel *ex vivo* model, we investigated the impact of RBC eNOS on erythrocrine function and vascular homeostasis.
• RBCs from eNOS knockout mice induce endothelial dysfunction in healthy vessels, due to the induction of an imbalance between NO and ROS within the vascular microenvironment.
• Similar to eNOS knockout mice, RBCs from patients with preeclampsia induce endothelial dysfunction, which was not observed by RBC from women with gestational hypertension.
• These findings provide a better understanding of the crucial role of RBC NO and ROS balance in mediation of cardiovascular disease pathogenesis.
• Targeting RBC NO homeostasis could be a novel therapeutic approach for endothelial dysfunction-related cardiovascular diseases.

## Graphical abstract

**Figure:**
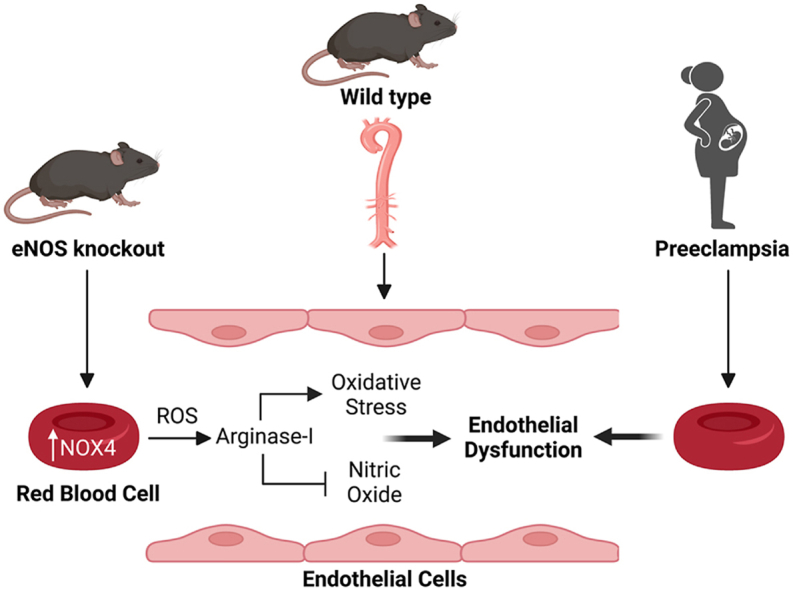

## 1 Introduction

Nitric oxide (NO) is a short-lived gaseous signalling molecule, which is crucial in the maintenance of cardiovascular homeostasis via stimulation of vasodilation and inhibition of both platelet aggregation and leukocyte adhesion [1].

Dysregulation of NO bioavailability is associated with multiple cardiometabolic pathologies, including hypertension, coronary artery disease, ischemia-reperfusion injury and atherosclerosis, as well as type 2 diabetes [2,3]. Canonically, under normoxic conditions, NO is synthesised via NO synthase (NOS) enzymes. Endothelial NOS (eNOS) is constitutively expressed in the vasculature and is dominant in the regulation of basal blood pressure (BP) via its autocrine and paracrine actions [1,4]. Under hypoxic conditions, NOS-dependent NO synthesis is greatly compromised, leading to decreased NO formation and potentially also eNOS uncoupling with increased generation of reactive oxygen species (ROS) [5]. It has been proposed that eNOS competes with arginase for the common substrate l-arginine and the dynamic interplay between these two enzymes can influence NO bioavailability, as well as ROS generation [6].

In the circulation, red blood cells (RBCs) are the most abundant cell type and serve to shuttle O_2_ and CO_2_ between the lungs and systemic tissues via interactions with intracellular haemoglobin (Hb). The close proximity of RBCs with adjacent vascular beds facilitates their dynamic interaction, which maintains vascular integrity and function. Additionally, RBCs possess the capacity to synthesise, store and export bioactive molecules, including NO-like bioactivity, which in turn may affect signaling in endothelial cells and smooth muscle cells [7,8]. RBCs have been demonstrated to induce NO-dependent vasorelaxation under hypoxia [[9], [10], [11]]. However, due to the ultrarapid NO scavenging by oxyhemoglobin (oxyHb), the functional importance of RBC export of NO has long been debated. Proposed sources of RBC-derived NO bioactivity thus far include S-nitrosated Hb (SNO-Hb) [8], nitrosyliron(II)hemoglobin (HbFe(II)NO) [12], mobile nitrosyl-heme [13], and nitrite [9,14,15]. In addition, release of RBC-derived ATP may also occur under conditions of shear stress or hypoxia induced vasodilatation [16,17].

Evidence supporting a physiological role for an intrinsic RBC eNOS is now mounting, showing evidence for eNOS expression in the RBC, but the signalling mechanisms and its functional importance is still debated [18]. More recently, several reports have supported the presence of a catalytically active RBC eNOS, localised to the plasma membrane and cytoplasm [18,19], which influences circulating nitrite levels [20], blood pressure [20,21] and mediates cardioprotection in ischemia-reperfusion injury [22]. In addition, arginase-1 (Arg1) expression/activity has been demonstrated in RBCs which can modulate RBC eNOS-derived export of NO bioactivity [22]. This has been shown in RBCs from type 2 diabetic [[23], [24], [25], [26]] and pre-eclamptic patients [27] *ex vivo*.

Although studies thus far have highlighted the link between dysregulation of RBC homeostasis and cardiometabolic pathologies *per se*, mechanistic knowledge on the influence of eNOS activity on RBC and vascular endothelium interaction is still lacking. In the present study we aimed to investigate the role of RBC eNOS in regulation of vascular function. Specifically, we hypothesized that RBC-derived NO bioactivity is crucial for maintenance of vascular function and that lack of RBC eNOS may induce endothelial dysfunction. Utilising an *ex vivo* approach to specifically investigate erythrocrine function, we aimed to dissect the mechanisms underlying this interaction.

## 2 Materials and methods

The data that support the present study are available from the corresponding author upon reasonable request.

### 2.1 Ethical approval

This study was approved by the regional Institutional Animal Care and Use Committee and performed according to the US National Institutes of Health guidelines (NIH publication NO. 85-23, revised 1996) and EU directive 2010/63/EU for the conduct of experiments in animals. The regional ethics committee in Stockholm, Sweden approved collection of clinical blood samples (Dnr: 2020-01596). Prior to inclusion, all study participants provided written informed consent. All procedures were conducted according to the updated Declaration of Helsinki.

### 2.2 Animals and tissue

Commercially available conventional male and female wildtype C57BL/6J (Janvier Labs) and.

C57BL/6J/B6.129P2-*Nos3*^*tm1Unc*^/J homozygous eNOS^-/-^ (eNOS KO; Jackson Laboratory, USA) mice were used. Basal vascular NO production was not detected in eNOS KO mice (Fig. S1). Conditional endothelial cell-specific Arg1 KO mice (EC-Arg1 KO) were generated as described elsewhere [21]. Briefly, homozygous Arg^lox/flox^ mice were crossed with Cdh5-Cre/ERT2^pos^ mice to obtain Arg1^lox/flox^ Cdh5-Cre/ERT2^pos^ and Arg1^lox/flox^ Cdh5-Cre/ERT2neg mice. To induce EC-specific activation of the Cre-recombinase, Cre positive and negative mice were treated with tamoxifen (TAM, 33 mg/kg/day) for 5 consecutive days and allowed a 21-day waiting period after the last injection, which generated EC Arg1 KO (EC Arg1^lox/flox^ Cdh5-Cre/ERT2^pos^ + TAM) mice and their respective Cre negative WT littermate controls (EC Arg1^lox/flox^ Cdh5-Cre/ERT2^neg^ + TAM). All mice used in this study were housed and acclimatized for at least one month in a temperature and humidity-controlled environment, kept in a 12:12-h light-dark cycles and fed with standard rodent chow and water *ad libitum,* in the animal facility of Comparative Medicine, Karolinska Institutet. On the day of the experiments, the mice were between 3 and 6 months old.

### 2.3 Clinical sampling

#### 2.3.1 Study population

14 preeclamptic, and 4 gestationally hypertensive women receiving pre-natal care at the Women’s Clinic, Danderyd Hospital, Stockholm, were recruited in 2022. Diagnoses were according to current ACOG guidelines [28], from gestational week 20, who did not require imminent delivery. For controls, 5 healthy pregnant women (>gestational week 20) were recruited.

#### 2.3.2 Venous blood sampling

Venous blood was sampled using a BD sodium heparin vacutainer (1245647; BD Biosciences, Stockholm, Sweden), placed on regular ice (+4°C) and immediately transported to Biomedicum Karolinska Institute for experimental analyses. Following delivery of blood samples in BD sodium heparin vacutainers, 400uL of whole blood was immediately snap-frozen in a 1 mL syringe and stored in liquid nitrogen until EPR analyses. Next, blood components were isolated (900×*g*, 5 min, 4°C), and RBCs were washed as described below (section 2.4).

### 2.4 Experimental protocol

**Fig. 1:**
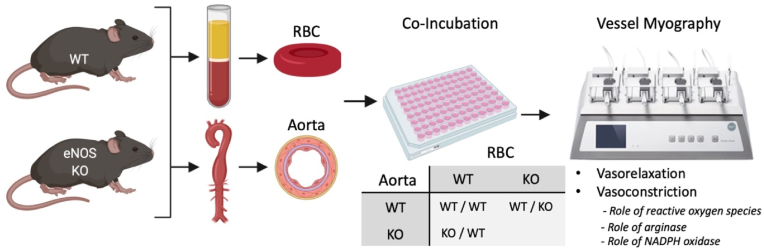
**Overview of experimental protocol.** Aortae and whole blood were isolated from eNOS WT and global eNOS KO mice. Whole blood constituents were separated, and red blood cells (RBC) were washed. Aortae were cleaned and separated into aortic rings in PSS. In indicated combinations, aortic rings and washed RBCs (10% haematocrit) were co-incubated (37 °C; 5% CO_2_) overnight (18 h). Following incubation, aortic rings were washed, and vessel reactivity (vasorelaxation and contractility) was assessed via vascular wire myography. To further assess the role of reactive oxygen species (ROS), arginase and NADPH oxidase (NOX) activity on the observed functional interaction between eNOS KO RBCs and WT aortae, the co-incubations were simultaneously treated with TEMPOL, nor-NOHA and NOX inhibitors, respectively.

The design of the experimental *ex vivo* approach used in the present study is highlighted in Fig. 1.

### 2. 5 Tissue preparation

Mice were anesthetized with isoflurane inhalation and blood was sampled via the inferior vena cava into 1.5 mL Eppendorf tubes containing 0.5 mmol EDTA (Sigma-Aldrich, Stockholm, Sweden). Whole blood was immediately centrifuged at 4°C for 5 min (900×*g*) and plasma was collected and stored at -80°C for later analyses. In the remaining sample, the buffy coat was aspirated and discarded. PBS (500uL) was added to the remaining red blood cells (RBCs), mixed gently and centrifuged (500×*g*, 5 min, 4°C). PBS was aspirated and washing was repeated twice (3x total). Following blood sampling, descending thoracic aortae were immediately isolated and dissected into aortic rings (*n=*8–12 per mouse) in ice-cold physiological salt solution (PSS, composition in mmol/L: NaCl, 130; 8 KCl, 4.7; CaCl_2_, 1.6; KH_2_PO_4_, 1.18; MgSO_4_·7H_2_O, 1.17; NaHCO_3_, 14.9; glucose, 5.5; and EDTA, 0.026) and utilised for tissue co-incubation studies.

### 2. 6 Tissue co-incubation studies

The influence of eNOS activity of RBCs on vascular reactivity in murine aortic preparations was assessed. Washed RBC samples from WT or eNOS KO mice were diluted in high glucose DMEM (Gibco; 17.5 mM) to 10% haematocrit. Sample dilutions were incubated with murine aortic rings from either WT or eNOS KO mice in a 96-well plate at a final volume of 200uL and incubated overnight (18 h) in a tissue culture incubator at 37°C with 5% CO_2_. Naïve healthy aortic rings (Control) and eNOS KO aortic rings, without any RBCs, were also incubated in high glucose DMEM. The combinations of RBC-aortae incubations are illustrated in Fig. 1, for a single myography experiment all illustrated combinations were compared.

### 2. 7 Ex vivo vascular reactivity analyses using vascular myography

Aortic rings were repeatedly washed with PSS to remove RBCs and mounted onto a onto the pins of multi wire myograph system (Model 620 M; Danish Myo Technology, Denmark). The chambers were pre-filled with 8 mL of PSS solution (37°C, pH 7.4) aerated with carbogen (95% O_2_; 5% CO_2_). Isometric tension was recorded with Powerlab system (Powerlab 4/30). After mounting, vessels were equilibrated for 45 min. A loading force of 6 mN was added to the vessel to mimic the near physiological pressure. After another 45 min equilibration, vessels were contracted with KCl (120 mM) solution to determine the reactivity of the vascular smooth muscle cells. Vessels were then washed (3x) before performing concentration response curves. After washing, the aortic rings were pre-contracted with increasing concentrations of Phenylephrine (PE, 0.1 nM to 10 μM) to reach approximately 80% of KCl-induced contraction. After reaching a stable plateau phase, the endothelium-dependent relaxation was induced by a cumulative concentration of acetylcholine (ACh, 1 nM to 100 μM). For the endothelium-independent relaxation, a cumulative concentration-dependent response for sodium nitroprusside (SNP, 1 nM to 100 μM) was induced. Vessels with unstable preconstruction were excluded from the study.

### 2. 8 Role of arginase, nitric oxide, and oxidative stress on vascular function

To investigate the mechanistic impact of RBCs on vascular reactivity, treatments were included in the co-incubation preparations overnight (Fig. 1). To assess the role of arginase, NO, NADPH oxidase (NOX) and oxidative stress, the following pharmacological agents were included in the co-incubation preparations: the arginase inhibitor N-omega-hydroxy-nor-l-arginine (Nor-NOHA; 10 μM, Bachem), the slow release NO donor DetaNONOate (30 μM, Sigma), the superoxide dismutase mimetic 4-Hydroxy-TEMPO (TEMPOL; 1 mM, Merck) and the NOX inhibitors GLX7013114 (NOX4; 3 μM, Glucox Biotech AB, Stockholm), GLX481304 (NOX2/4; 30 μM, Glucox Biotech AB, Stockholm) and the iNOS specific inhibitor, 1400W (0.1 μM; abcam, UK). In separate experiments, after washing and mounting co-incubated vessels, TEMPOL (300 μM), the NOX4 inhibitor, GLX7013114 (3 μM) and the iNOS specific inhibitor, 1400W (0.1uM) were acutely added to the myograph chambers and incubated for 30 min prior to beginning the myography protocol. In addition, WT RBCs and eNOS KO RBCs were co-incubated overnight with aorta from conditional EC-Arg1 KO mice and their WT littermate controls, followed by the aforementioned myography protocol.

### 2. 9 In situ superoxide production of aortic rings

Following the aforementioned tissue co-incubations overnight, aortic rings were immediately washed with PSS, and subsequently stabilised in fresh PSS, at 37°C for 60 min. Next, rings were fixed in optical cutting temperature compound (OCT; Tissue-Tek® O.C.T.™) and immediately snap-frozen at -20°C. Rings were cryosectioned (8 μm) and collected using an NX70 cryostat (Thermo Scientific Inc., Runcorn, UK) and mounted on histological slides in duplicate. For each ring, one section was incubated with Dihydroethidium (DHE, 5 μM; Sigma-Aldrich, Germany) and another with PSS solution (control) in darkness for 15 min. After incubation, the sections were washed with deionized H_2_O twice (1 min). Following excitation by a xenon lamp, images (x10 magnification) were immediately captured using a Nikon fluorescence microscope (TE2000-U, Japan) at an emission wavelength of 570–645 nm (TRITC Filter), using a digital microscope camera (Nikon DS-5M, Japan) and quantified by ImageJ 2.0 software. The DHE-derived 2-OH-E+ levels (A.U) represent the difference between the incubation with PSS and DHE.

### 2. 10 EPR analyses

EPR data were expressed in arbitrary units. All EPR spectra were obtained utilising a bench top Magnettech MiniScope MS5000 ESR spectrometer (Freiberg Instruments), recordings were made at 77K using a Dewar flask (Wilmad, USA).

#### 2. 10.1 NO spin trapping in mouse aortas

Aortas obtained either from WT or eNOS KO mice were stimulated with Ca-ionophore A23187 (10 μM; 1hr; 37°C) in the presence of NO trap, colloid Fe(II)- diethyldithiocarbamate, Fe(DETC)2, as described previously [29]. EPR spectra were recorded at 77K using an X-band spectrometer MS5000 (Magnettech-Bruker). EPR parameters were 10 mW of microwave power, 1 mT of amplitude modulation, 100 kHz of modulation frequency, 60 s of sweep time and 4 number of scans. The results were expressed in EPR arbitrary units.

#### 2. 10.2 RBC NO-heme levels

Venous blood was centrifuged, unwashed RBC samples (200 μL) were placed in 1 ml end-cut syringes, then frozen and stored in liquid nitrogen. The EPR measurements were carried out at 77K using an X-band spectrometer MS5000 (Magnettech-Bruker). The instrument settings were as follows: 10 mW microwave power, 0.5 mT amplitude modulation, 100 kHz modulation frequency, 336 mT center field, 12 mT sweep width, 90 s sweep time and 16 scans. The assessments of 5-coordinated NO-heme levels were performed by the measurement of the amplitude of 1st (lower field) component of the characteristic triplet EPR signal [30].

#### 2. 10.3 Cu^2+^-ceruloplasmin and Fe^3+^-transferrin in blood

Whole blood samples (0.5 ml) were placed in 1 ml end-cut syringes and frozen in liquid nitrogen. The EPR measurements were carried out at 77K using an X-band spectrometer MS5000 (Magnettech-Bruker). The instrument settings were as follows: 10 mW microwave power, 0.8 mT amplitude modulation, 100 kHz modulation frequency, 175 mT center field, 350 mT sweep width, 300s sweep time and 1 scan. The Cu^2+^-ceruloplasmin and Fe^3+^-transferrin levels were assessed by the measurement of amplitude of the EPR signal at g = 4,3 and g = 2.05, respectively [31].

### 2. 11 Statistical analyses

Data were analysed using Graphpad Prism (version 6.0, Graphpad Software, UK). Data expressed as mean ± SEM unless otherwise indicated. Individual vessel concentration-response curves were determined by nonlinear regression analysis and analysed with 2-way ANOVA with repeated measurements and Tukey *post-hoc* testing. For multiple comparisons of other parameters, a 1-way ANOVA adjusted with Tukey’s correction was utilised. For comparison between two groups (*i.e.,* arginase activity) data was analysed with unpaired Student’s t-test. Statistical significance was defined as p<0.05.

## 3 Results

### 3.1 RBCs from eNOS KO mice induce endothelial dysfunction

**Fig. 2:**
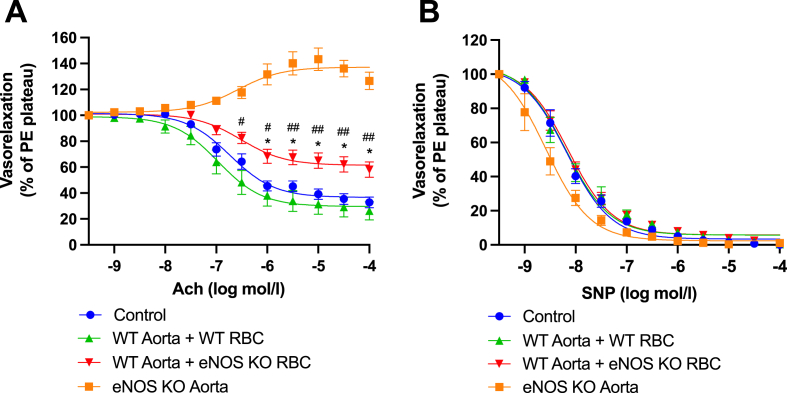
**RBCs from eNOS KO mice induce endothelial dysfunction. (A)** Endothelial-dependent vasorelaxation (% of Phenylephrine plateau; PE) response curve to acetylcholine (Ach; log mol/L) and **(B)** endothelial-independent vasorelaxation (% of phenylephrine plateau; PE) response curve to nitroprusside (SNP) of mouse aortic rings following overnight incubation with Control, RBC from a WT mouse (WT RBC) or RBC from an eNOS KO mouse (eNOS KO RBC). Control and eNOS KO aorta incubation denotes incubation of a WT or eNOS KO aorta, respectively, with DMEM media without RBC. Data expressed as mean ± SEM; Control, n = 7; WT aorta + WT RBC, n = 17; WT aorta + eNOS KO RBC, n = 18; eNOS KO aorta; n = 10, analysed via ordinary 2-way ANOVA with multiple comparisons and Tukey’s post-hoc test. Statistical significance defined as *p < 0.05, **p < 0.01; ***p < 0.005; ****p < 0.001; Comparisons of WT aorta + eNOS KO RBC *vs Control; # vs WT aorta + WT RBC; † vs eNOS KO aorta.

To assess the erythrocrine function of RBCs from eNOS KO mice, we co-incubated WT aortas + RBC isolated from eNOS KO mice overnight (18 h), then washed the aortae and subsequently assessed their function. A significant reduction in endothelial-dependent relaxation (EDR) was observed, compared with WT vessels co-incubated with WT RBCs (Fig. 2A). Endothelium independent relaxation was unaffected (EIR; Fig. 2B). These findings were mirrored in separate experiments utilising RBCs from a different strain of global eNOS KO mice (DelCre eNOS KO mice) and their respective WT controls (eNOS^flox/flox^; Fig. S3) as well as from female mice (Fig. S4). These data evidence the presence of a dysfunctional crosstalk between the healthy endothelium and RBCs from eNOS KO mice, which is not sex or strain-specific, suggesting that RBC eNOS *per se* may be specifically required for maintenance of vascular endothelial function.

### 3.2 Dysregulated NO and ROS homeostasis underlie eNOS KO RBC-induced endothelial dysfunction

**Fig. 3:**
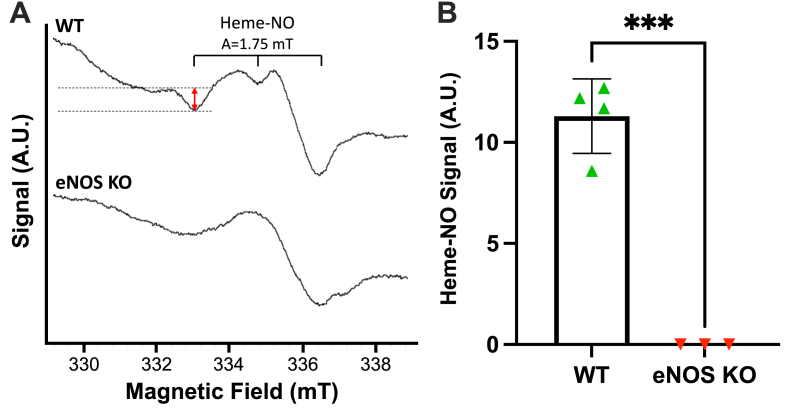
**Heme-NO signal is not detected in RBCs from eNOS KO mice. (A)** EPR spectra and **(B)** quantification of Heme-NO signal in mouse RBCs. Spectra were recorded at 77K. Instrument settings indicated in methods. Analysed via a Students *t*-test for unpaired observations. Statistical significance defined as * *p* < 0.05, ***p < 0.005.

**Fig. 4:**
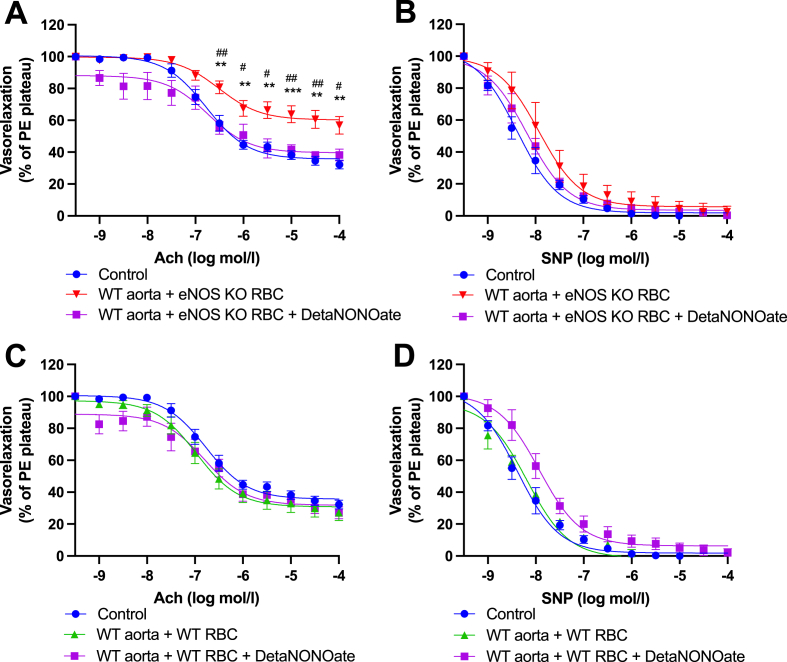
**Functional role of dysregulated NO homeostasis in eNOS KO RBC-induced endothelial dysfunction. (A, C)** Endothelial-dependent vasorelaxation (% of Phenylephrine plateau; PE) response curve to acetylcholine (Ach; log mol/L) and **(B, D)** endothelial-independent vasorelaxation (% of phenylephrine plateau; PE) response curve to nitroprusside (SNP) of WT mouse aortic rings following overnight incubation with Control, **(A, B)** RBC from an eNOS KO mouse (eNOS KO RBC) **(C, D)** RBC from a WT mouse (WT RBC) ± Deta-NONOate (30 μM). Control incubation denotes incubation with DMEM media without RBC. Data expressed as mean ± SEM; n = 6–7 per group; analysed via ordinary 2-way ANOVA with multiple comparisons and Dunnetts post-hoc test. Statistical significance defined as, *p < 0.05, **p < 0.01; ***p < 0.005; Comparisons of WT aorta + eNOS KO RBC *vs Control; # vs WT aorta + eNOS KO RBC + Treatment.

Next, we aimed to elucidate whether a lack of eNOS-derived NO bioactivity account for the RBC-induced ED phenotype. RBCs from eNOS KO mice exhibited a significantly decreased Heme-NO signal vs RBCs from WT mice (Fig. 3), indicating eNOS-dependent levels of bound NO in RBCs. Next, we co-incubated WT aortae and eNOS KO RBCs with the slow releasing NO donor DetaNONOate (t_1/2_ 20 h) overnight (Fig. 4). We observed significantly improved EDRs in DetaNONOate treated vs non-treated eNOS KO RBC co-incubated vessels, to levels comparable to control (Fig. 4A). EIR responses remained unchanged in all the experimental groups (Fig. 4B, D). These data suggest RBC-derived NO bioactivity is a key regulator of adjacent vascular endothelial function.

**Fig. 5:**
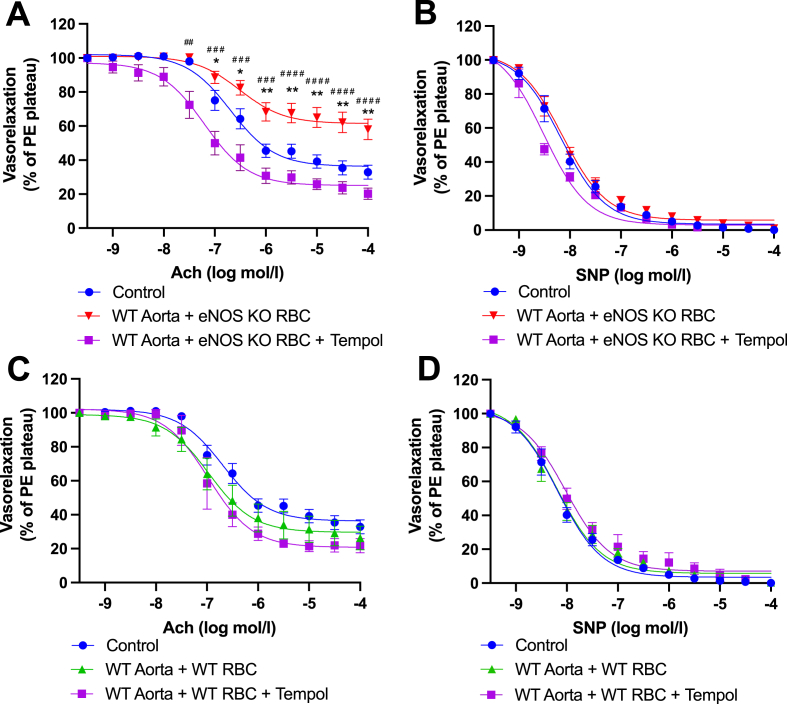
**Functional role of ROS in eNOS KO RBC-induced endothelial dysfunction. (A, C)** Endothelial-dependent vasorelaxation (% of Phenylephrine plateau; PE) response curve to acetylcholine (Ach; log mol/L), **(B, D)** endothelial-independent vasorelaxation (% of phenylephrine plateau; PE) response curve to nitroprusside (SNP) of WT mouse aortic rings following overnight incubation with Control, **(A,B)** RBC from an eNOS KO mouse (eNOS KO RBC), **(C, D)** RBC from a WT mouse (WT RBC), ± TEMPOL (1 mM). Control incubation denotes incubation with DMEM media without RBC. Data expressed as mean ± SEM; n = 6–8 per group; analysed via ordinary 2-way ANOVA with multiple comparisons and Dunnetts post-hoc test. Statistical significance defined as **p < 0.05, **p < 0.01; ***p < 0.005; ****p < 0.001; Comparisons of WT aorta + eNOS KO RBC *vs Control; # vs WT aorta + eNOS KO RBC + TEMPOL; † vs eNOS KO aorta.

A small number of studies have reported RBC iNOS expression via immunolabelling in healthy rats [32] and humans [33]. To investigate whether iNOS was perturbed in the observed ED phenotype, we first included an iNOS specific inhibitor (1400W) with the overnight RBC-aortae co-incubations. We did not observe any beneficial effect of iNOS inhibition on EDRs, as evidenced by comparable EDRs between WT aorta + eNOS KO RBC with vs without 1400W (Fig. S4A). Aortae incubated with RBCs from WT mice exhibited comparable EDRs across all groups (Fig. 5C), EIR responses for all groups remained comparable (Figs. S4B and D). Similarly, acute incubation of 1400W to the myograph chambers following eNOS KO RBC exposure did not improve EDRs (Fig. S5A) and had also no effect on EIRs (Fig. S5B). Acute incubation with 1400W did not impact EDR responses or EIRs of aortae incubated with WT RBCs (Fig. S5 C, D).

**Fig. 6:**
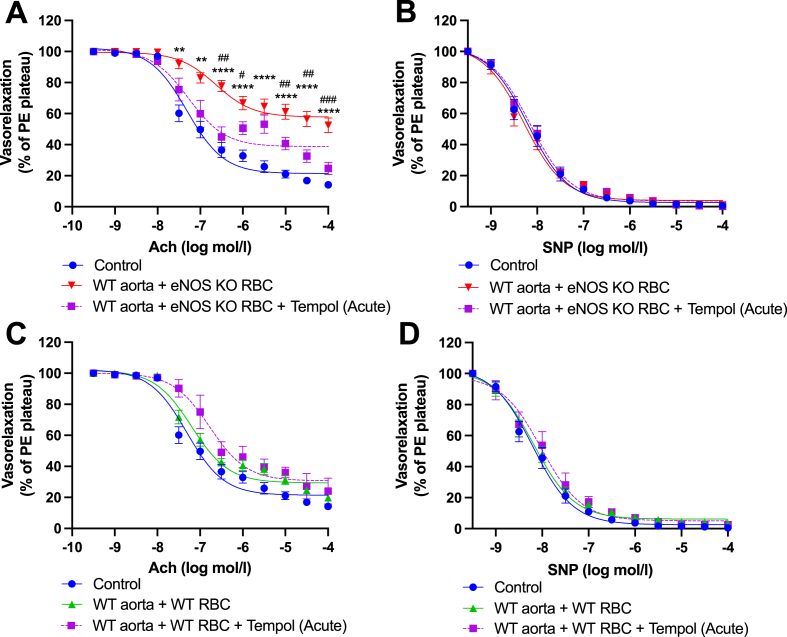
**Functional role of vascular ROS in eNOS KO RBC-induced endothelial dysfunction. (A, C)** Endothelial-dependent vasorelaxation (% of Phenylephrine plateau; PE) response curve to acetylcholine (Ach; log mol/L) and **(B, D)** endothelial-independent vasorelaxation (% of phenylephrine plateau; PE) response curve to nitroprusside (SNP) of WT mouse aortic rings following overnight incubation with Control, **(A, B)** RBC from an eNOS KO mouse (eNOS KO RBC), **(C, D)** RBC from a WT mouse (WT RBC) with TEMPOL (300 μM) added acutely to the myograph chambers and incubated for 30 min prior to beginning the myography protocol. Control incubation denotes incubation with DMEM media without RBC. Data expressed as mean ± SEM; n = 6–8 per group. Analysed via ordinary 2-way ANOVA with multiple comparisons and Dunnetts post-hoc test. Statistical significance defined as, *p < 0.05, **p < 0.01; ***p < 0.005; ****p < 0.001; Comparisons of eNOS KO RBC *vs Control; # vs eNOS KO RBC + Acute TEMPOL.

A delicate balance between NO bioavailability and levels of ROS is required to maintain endothelial function. Therefore, we next aimed to investigate whether the observed disruption of RBC-derived NO bioactivity from eNOS KO mice impacted ROS generation in our model. First, the radical scavenger TEMPOL, which has superoxide dismutase and catalase activities, was added to overnight co-incubation preparations. TEMPOL significantly preserved the EDRs of WT aortae co-incubated with eNOS KO RBCs, with responses comparable to controls (Fig. 5A). In all groups, EIRs were comparable, and without any effect of TEMPOL (Fig. 5B). In WT control aortae, overnight co-incubation with WT RBCs and simultaneous treatment with TEMPOL, had no effect on EDR or EIR (Fig. 5C, D). When TEMPOL was added acutely to the myograph chambers of co-incubated aortae, EDRs of WT aortae co-incubated with eNOS KO RBCs were significantly improved compared to vessels without acute TEMPOL treatment (Fig. 6A). Again, TEMPOL had no effect on aortic EDRs co-incubated with WT RBCs (Fig. 6C). EIRs of WT aortic rings, co-incubated with WT or eNOS KO RBCs, were comparable to controls (Fig. 6D).

**Fig. 7:**
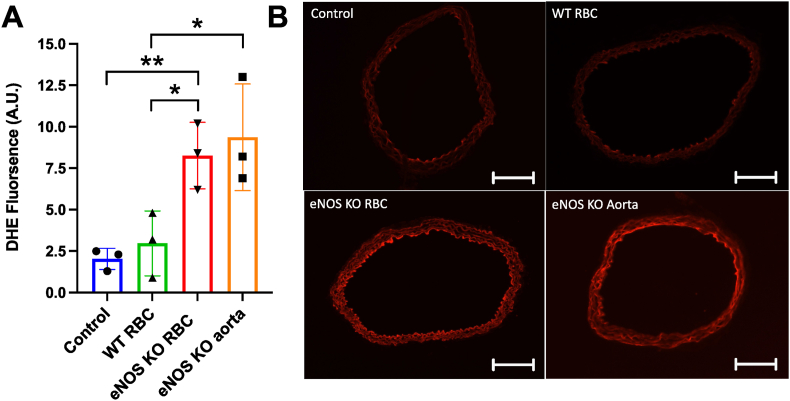
**eNOS KO RBCs induce elevated vascular superoxide production measured by DHE fluorescence. (A)** Fluorescence intensity of WT mouse aortic rings following overnight incubation with RBC from an eNOS KO mouse (eNOS KO RBC) or RBC from a WT mouse. Control and eNOS KO aorta incubation denotes incubation of a WT or eNOS KO aorta, respectively, with DMEM media without RBC. Fluorescence quantified by optic densitometry (arbitrary units). **(B)** Representative images (x10 magnification) of aortic ring sections incubated in the presence DHE. Red fluorescence produced when DHE is oxidised to 2-hydroxyethidium by O_2_^•−^. Scale bar represents 200 μm in all panels. Data expressed as mean ± SEM; n = 3 per group. Statistical significance defined as *p<0.05; **p < 0.01 between indicated groups.

Accordingly, superoxide (O_2_^−^) levels, as measured by DHE fluorescence, were significantly elevated in WT aortae co-incubated with RBCs from eNOS KO vs WT mice (Fig. 7). Of note, aortae exposed to eNOS KO RBCs exhibited comparable DHE fluorescence levels to eNOS KO aortas (Fig. 7). These data suggest an induction of oxidative stress within the endothelium as a result of exposure to RBCs lacking eNOS, and that this increase in ROS generation is maintained following removal of the eNOS KO RBCs.

**Fig. 8:**
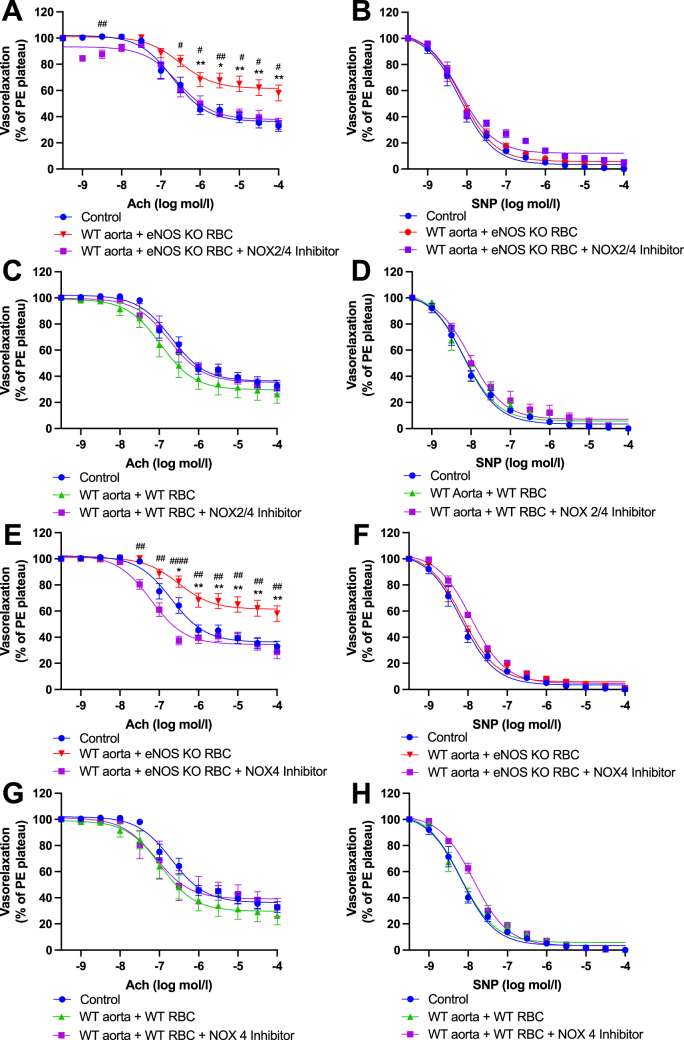
**Functional role of dysregulated elevated ROS in eNOS KO RBC-induced endothelial dysfunction. (A, C, E, G)** Endothelial-dependent vasorelaxation (% of Phenylephrine plateau; PE) response curve to acetylcholine (Ach; log mol/L) and **(B, D, F, H)** endothelial-independent vasorelaxation (% of phenylephrine plateau; PE) response curve to nitroprusside (SNP) of WT mouse aortic rings following overnight incubation with Control, RBC from an **(A, B)** eNOS KO mouse (eNOS KO RBC) or **(C, D)** WT mouse ± NOX 2&4 inhibitor (30 μM) or **(E, F)** an eNOS KO mouse (eNOS KO RBC) or **(G, H)** WT mouse ± NOX 4 inhibitor (3 μM). Control incubation denotes incubation with DMEM media without RBC. Data expressed as mean ± SEM; n = 10–15 per group; analysed via ordinary 2-way ANOVA with multiple comparisons and Dunnetts post-hoc test. Statistical significance defined as *p < 0.05, **p < 0.01; Comparisons of WT aorta + eNOS KO RBC *vs Control; # vs WT aorta + eNOS KO RBC + Treatment.

NOXs are considered major sources of ROS generation in the arterial wall, and oxidative stress due increased NOX expression/activity has been associated with aging and cardiometabolic disorders, including hypertension and diabetes [34]. To determine whether NOXs were the source of the ROS influencing EDR in the present model, in separate experiments we included pharmacological inhibitors of NOX; a combined NOX2&4 inhibitor and a selective NOX4 inhibitor to overnight co-incubation preparations (Fig. 8). Simultaneous inhibition of NOX2&4, and sole inhibition of NOX4 both prevented eNOS KO RBC-induced endothelial dysfunction in WT aortae (Fig. 8A, E, respectively), but had no impact on EDR on WT vessel co-incubated with WT RBCs (Fig. 8C, G). Inhibition of the same NOX isoforms did not alter EIR responses in any of the experimental groups, i.e., remaining comparable to controls (Fig. 8B, D, F & H). These data suggest that NOX4 is a key mediator of the observed eNOS KO RBC-induced ED.

**Fig. 9:**
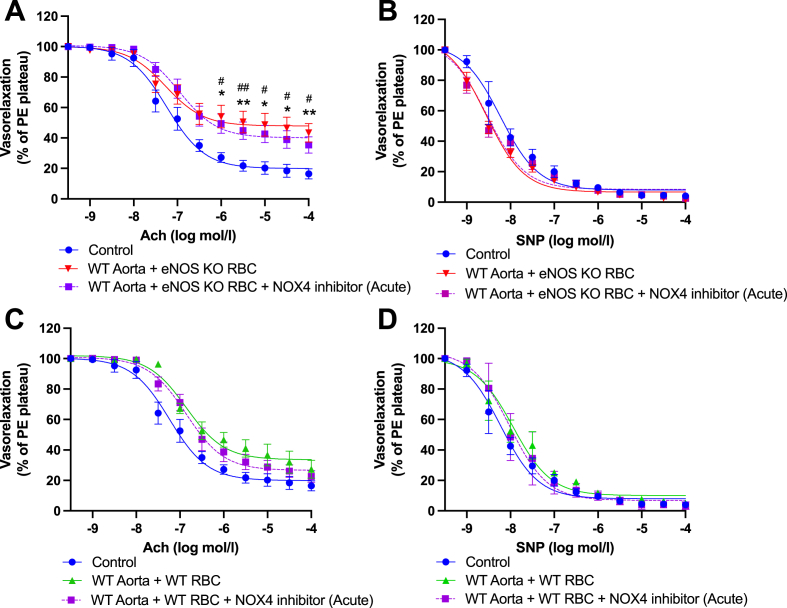
**Vascular NOX4 not involved in eNOS KO RBC-induced endothelial dysfunction. (A, C)** Endothelial-dependent vasorelaxation (% of Phenylephrine plateau; PE) response curve to acetylcholine (Ach; log mol/L) and **(B, D)** endothelial-independent vasorelaxation (% of phenylephrine plateau; PE) response curve to nitroprusside (SNP) of WT mouse aortic rings following overnight incubation with Control, RBC from an **(A, B)** eNOS KO mouse (eNOS KO RBC) or **(C, D)** WT mouse ± NOX4 inhibitor (3 μM) added acutely to the myograph chambers and incubated for 30 min prior to beginning the myography protocol. Control incubation denotes incubation with DMEM media without RBC. Data expressed as mean ± SEM; n = 5–15 per group; analysed via ordinary 2-way ANOVA with multiple comparisons and Dunnetts post-hoc test. Statistical significance defined as *p < 0.05, **p < 0.01; comparisons of Control vs *WT aorta + eNOS KO RBC; # vs WT aorta + eNOS KO RBC + NOX4 inhibitor.

To determine whether NOX4 was acting at the RBC or vascular level, following RBC-vessel co-incubations, we added a NOX4 inhibitor acutely to the myograph chambers. Interestingly, acute inhibition of vascular NOX4 did not significantly improve endothelial function, as evidenced by comparable EDRs between WT aorta + eNOS KO RBCs with vs without acute NOX4 inhibition (Fig. 9A). Acute NOX4 inhibition had no functional impact on EDRs or EIRs of all experimental groups (Fig. 9A, B, C, D).

Taken together, these data suggest that in the absence of eNOS, RBC NOX4 is a source of elevated ROS, such that RBC eNOS-derived NO bioactivity is crucial in preventing an overabundance of, in part, RBC NOX4-dependent ROS production, and in turn, endothelial dysfunction.

### 3.3 Vascular arginase 1 is involved in eNOS KO RBC-induced endothelial dysfunction

**Fig. 10:**
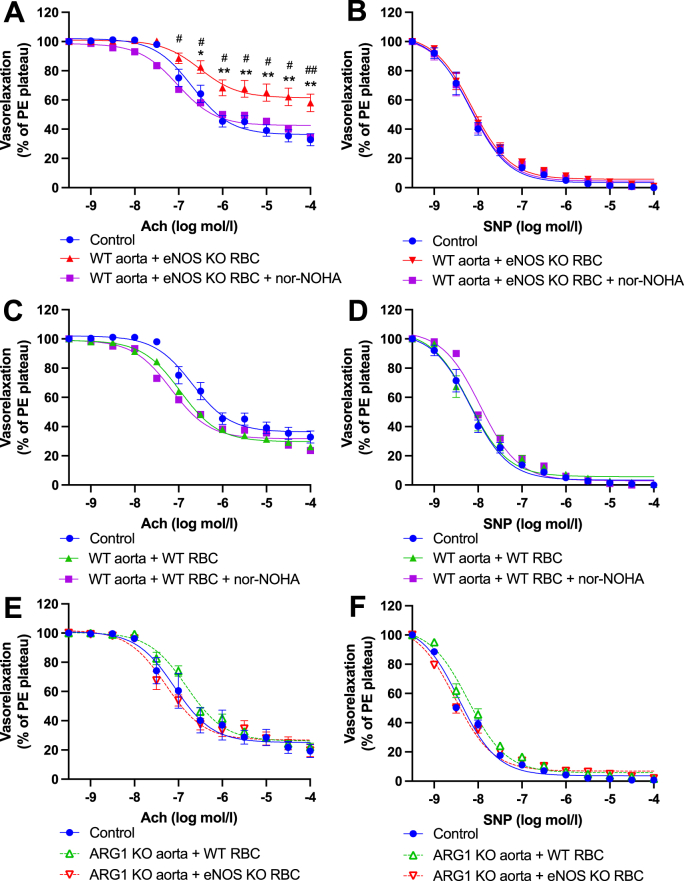
**Functional role of dysregulated Arginase in eNOS KO RBC-induced endothelial dysfunction. (A, C, E)** Endothelial-dependent vasorelaxation (% of Phenylephrine plateau; PE) response curve to acetylcholine (Ach; log mol/L) and **(B, D, F)** endothelial-independent vasorelaxation (% of phenylephrine plateau; PE) response curve to nitroprusside (SNP) of mouse aortic rings. **(A, B, C, D)** WT mouse aortic rings following overnight incubation with Control, **(A, B)** RBC from an eNOS KO mouse (eNOS KO RBC) **(C, D)** RBC from a WT mouse (WT RBC) ± Nor-NOHA (10 μM). **(E, F)** Endothelial-specific Arginase 1 KO mouse aortic rings following overnight incubation with Control, RBC from a WT mouse (WT RBC) or RBC from an eNOS KO mouse (KO RBC; 10%). Control incubation denotes incubation with DMEM media without RBC. Data expressed as mean ± SEM; n = 9–11 per group; analysed via ordinary 2-way ANOVA with multiple comparisons and Dunnetts post-hoc test. Statistical significance defined as *p < 0.05, **p < 0.01; Comparisons of aorta + eNOS KO RBC *vs Control; # vs eNOS KO RBC + Treatment.

In both endothelial cells and RBCs, Arginase 1 competes with eNOS for the common substrate l-Arginine. In pathological states, the balance between the activity of these enzymes is disrupted, whereby elevated arginase activity shunts l-arginine into ornithine and urea production, and in turn, reduces eNOS-derived NO formation and eNOS uncoupling. Importantly, arginase activity is stimulated by and results in an overabundance of ROS, thus contributes to the propagation of oxidative stress. Following this line of enquiry, we next focused on the influence of arginase in the observed eNOS KO RBC-induced endothelial dysfunction. First, we co-incubated RBC-aortae preparations with the arginase inhibitor; Nor-NOHA. Pharmacological arginase inhibition prevented eNOS KO RBC-induced endothelial dysfunction (Fig. 10A) but had no impact on aortae co-incubated with WT RBC (Fig. 10C). EIR was again unaffected (Fig. 10B, D).

To address whether Arginase 1 is directly involved at the vascular level, we next utilised a genetic approach whereby aortae from endothelial cell-specific Arg1 KO mice (EC-Arg1 KO) were co-incubated with RBCs isolated from eNOS KO mice. When RBCs from eNOS KO mice were incubated with EC-Arg1 KO aortae, endothelial function remained comparable to controls and WT RBC incubated aortae (Fig. 10E). Again, EIRs were unaffected (Fig. 10F). To compare arginase activity at the RBC level, we quantified arginase activity in the membrane fractions of RBCs from eNOS KO vs WT mice. No significant difference in RBC arginase activity was observed between groups (Fig. S6).

Together, these data suggest that RBC eNOS-derived NO bioactivity is protective against elevations in vascular arginase activity of adjacent vessels, such that a lack of RBC eNOS stimulates vascular arginase-dependent induction of endothelial dysfunction. Further, that a lack of RBC eNOS does not impact RBC arginase activity.

### 3.4 Close eNOS KO RBC-vascular proximity is required for induction of endothelial dysfunction

We next aimed to assess the spatial dynamics of the observed RBC-endothelial communication. First, we incubated washed RBCs (WT or eNOS KO) with DMEM media overnight, and subsequently collected the supernatants for another round of overnight co-incubation with WT aortae, followed by analysis of vascular function via myography. No differences in EDRs (Fig. S7C) or EIRs (Fig. S7D) were observed between Control, WT RBC and eNOS KO RBC groups. Next, we utilised a transwell insert (pore size: 0.4 μM) within the overnight the RBC-vessel co-incubations, then assessed vascular function. When direct contact between RBCs and aortae was prevented, both EDRs and EIRs were comparable across all groups (Figs. S7A and B). Together, these data suggest that the detrimental signal transferred between the eNOS KO RBCs and endothelium is rather short-lived and requires close proximity.

### 3.5 Products of haemolysis do not contribute to eNOS KO RBC-induced endothelial dysfunction

Scavenging of NO via cell-free heme was reported to occur ∼1000-fold faster than intracellular heme [35]. We aimed to examine the relationship between RBC eNOS, haemolysis and endothelial dysfunction in our model.

First, we assessed the degree of haemolysis which could induce the same degree of endothelial dysfunction in our model. Lysed WT (Fig. S8A) and eNOS RBCs (Fig. S8B), with a known % haemolysis, were co-incubated overnight with WT aortic rings and subsequently vascular reactivity was assessed. Endothelial dysfunction was observed in aortic rings which were incubated with 1.5% haemolysed RBCs, with no difference between maximal ACh-induced vasorelaxation between lysed WT and eNOS KO RBCs at each % lysis (Fig. S8C). Cyanmethemoglobin was quantified in the same RBC lysates (mg/ml; Fig. S8C).

Next, we examined the level of haemolysis in our *ex vivo* model. In separate experiments, supernatants were collected following overnight incubation of WT or eNOS KO RBC (10% hematocrit), and cyanmethemoglobin was quantified (mg/ml; Fig. S8D). No significant difference in cyanmethemoglobin (mg/ml) was observed between WT and eNOS KO RBC supernatants following overnight co-incubation. Further, the levels of cyanmethemoglobin in supernatant did not approach those required to induce endothelial dysfunction (Fig. S8C; 1.5% RBC lysates). These data suggest that eNOS KO RBCs are not prone to elevated haemolysis vs WT RBCs, and therefore products of haemolysis, such as cell-free heme, are unlikely to play a role in the observed deleterious interaction between eNOS KO RBC and the endothelium.

### 3.6 RBCs from PE patients induce endothelial dysfunction, whereas RBCs from GH patients do not

RBC eNOS activity and expression was demonstrated to clinically mirror endothelial function in a small cohort of CAD patients [36]. Further, in similar *ex vivo* models, RBCs from patients with cardiometabolic diseases associated with dysregulated eNOS function, including type 2 diabetes [[23], [24], [25]] and pre-eclampsia [27], were shown to induce an endothelial dysfunction mirroring that observed in the present study. Given these findings, we next aimed to evaluate the impact of donor RBCs from healthy pregnancy (HP), PE and gestationally hypertensive (GH) women in our model. Clinical characteristics of the study population are listed in Table S1. Gestational age at delivery (weeks), office SBP and DBP were significantly elevated in PE vs HP women. Office DBP was significantly elevated in GH vs HP women.

**Fig. 11:**
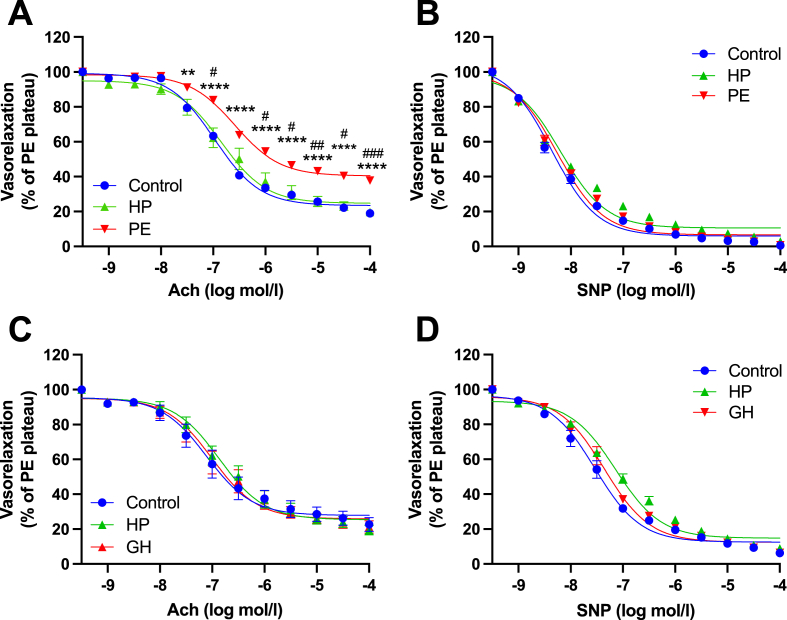
**RBCs from preeclamptic women, not gestationally hypertensive women, induce endothelial dysfunction. (A, C)** Endothelial-dependent vasorelaxation (% of Phenylephrine plateau; PE) response curve to acetylcholine (Ach; log mol/L) and **(B, D)** endothelial-independent vasorelaxation (% of phenylephrine plateau; PE) response curve to nitroprusside (SNP) of mouse aortic rings following overnight incubation with Control, RBC from healthy pregnant (HP) women and **(A, B)** preeclamptic women, or **(C, D)** gestationally hypertensive women. Control incubation denotes incubation of a WT aorta with DMEM media without RBC. Data expressed as mean ± SEM; biological replicates: HP, n = 5; PE, n = 14; GH, n = 4, experimental replicates: Control, n = 115; HP, n = 11; PE, n = 113; GH, n = 12. Analysed via an ordinary 2-way ANOVA with multiple comparisons and Tukey’s post-hoc test. Statistical significance defined as *p < 0.05, **p < 0.01; ****p < 0.001; Comparisons of PE*vs Control; # vs HP.

In mice aortae co-incubated with RBCs from PE women, we observed a significant reduction in EDR, compared with WT vessels co-incubated with RBCs from healthy pregnant (HP) women (Fig. 11A). Conversely, aortae co-incubated with RBCs from women with GH did not induce endothelial dysfunction, as evidenced by comparable EDR between control, HP, and GH groups (Fig. 11C). EIR were unaffected for all groups (Fig. 11B, D).

### 3.7 Ratio of Cu^2+^-ceruloplasmin:Fe^3+^-transferrin significantly elevated in whole blood from PE women

Within the systemic circulation, transferrin (TF) and ceruloplasmin (CP) are considered to contribute to redox regulation and inflammatory processes [37]. When unbound with transferrin (TF), iron catalyses oxidative damage and CP participates in the loading of TF with iron. Ferroxidase activity of CP is associated with anti-oxidative and anti-inflammatory activity by catalysing the oxidation of iron from the Fe^2+^ ion state to the Fe^3+^ ion state, a crucial step for adequate *trans*-cellular ionic transport. On the other hand, CP may elicit proinflammatory effects associated with the formation of hydroxyl radical from Fenton-type reactions of Cu^2+^ with hydrogen peroxide and the oxidation of low-density lipoprotein. Moreover, CP may decrease NO bioavailability through NO oxidase activity in blood [38], and increased levels has been associated with increased cardiovascular disease risk in clinical studies [39].

Considering the role of oxidative stress in the eNOS KO RBC-induced endothelial phenotype, we next aimed to characterize the level of systemic oxidative stress from the same cohort of PE and GH women via measuring modulators of oxidative damage and inflammation; Fe^3+^-TF and Cu^2+^-CP levels in whole blood via EPR. Characteristic EPR spectra from PE and HP blood samples are shown in Fig. S9A. The Fe^3+^-TF signal from whole blood from both PE and GH women was significantly decreased compared with healthy pregnant controls (Fig. S9B). The Cu^2+^-CP signal was significantly higher in blood from PE compared with GH women (Fig. S9C). The Cu^2+^-CP:Fe^3+^-TF ratio has been used in previous clinical studies as an index of oxidative stress related to iron metabolism [40]. When the ratio of Cu^2+^-CP:^3+^-TF was examined in each donor of this study, PE blood samples had significantly increased ration vs both GH and HP controls (Fig. S9D). The GH blood also had an elevated ratio, compared to healthy controls, but less pronounced compared to blood from patients with PE. Taken together, this indicates disturbance in blood antioxidant system and oxidative stress in PE, but to a less degree in GH.

## 4 Discussion

In the present study, we show that RBCs from global eNOS KO mice induce endothelial dysfunction in aortae from healthy WT mice *ex vivo*. Mechanistically, our findings suggest that this is attributed to the protective effect of RBC-derived NO bioactivity in preventing RBC NOX4-derived ROS inducing vascular oxidative stress, and in turn, endothelial dysfunction. Further, that the development of endothelial dysfunction via this process is dependent on vascular arginase. A similar abnormal RBC phenotype, inducing endothelial dysfunction *ex vivo*, was showed in patients with preeclampsia, which is a vulnerable group known to be associated with increased cardiovascular risk. Importantly, this was not observed in women with gestational hypertension, suggesting that this is not linked to elevated blood pressure *per se*.

Studies have demonstrated that dysregulation in RBC morphology, abundance and integrity, results in reduced RBC-derived NO-signalling, decreased oxygen transport capacity, and induction of a pro-oxidative and pro-inflammatory status, thereby playing an important role in endothelial homeostasis and significantly impacting cardiometabolic functions [18,41]. Evidence for a role of RBC eNOS in cardiovascular function is mounting. Indeed, decreased RBC eNOS expression and activity was evidenced in patients with coronary artery disease, which correlated with endothelial function [36]. *In vivo* experiments utilising chimeric RBCs from eNOS KO mice demonstrated significantly decreased levels of circulating NO metabolites (*i.e.,* nitrate and nitrite) and increased blood pressure vs controls [20]. In the same chimeras, cardioprotection against post-ischemia reperfusion was decreased, which was unaffected by NOS inhibition vs. controls [42]. More recently, utilising tissue specific eNOS KO mice, Leo et al. [21] highlighted a non-canonical role of RBC eNOS in regulation of blood pressure homeostasis *in vivo*. The authors observed a hypertensive phenotype in RBC-specific eNOS KO mice, as well as in endothelial-specific eNOS KO mice, although to a lesser degree in RBC eNOS KO mice. Moreover, RBC-specific KO mice exhibited decreased plasma nitrite and nitrate levels, and total NO species, concomitant with significantly decreased NO-heme, a marker of bound-NO, vs controls.

In the present study we aimed to directly investigate the mechanistic role of eNOS *per se* in RBCs in maintaining or modulating endothelial homeostasis and hence vascular function *ex vivo*. Recently, studies have provided insights into the mechanisms underlying the relationship between altered RBCs and the adjacent endothelium in specific pathologies, including dysregulated cardiac function [22]. In addition, RBCs from patients with type 2 diabetes have been demonstrated to induce endothelial dysfunction *ex vivo*. Evidenced underlying mechanisms include peroxynitrite-induced elevation of vascular arginase, subsequent modulation of NOS and ROS formation [[23], [24], [25]], and increased purinergic signalling in the vasculature [26]. Similarly, arginase- and ROS-dependent mechanisms were evidenced in preeclamptic-RBC-induced endothelial dysfunction *ex vivo* [27].

An intrinsic redox regulatory system is present within RBCs, which consists of both enzymatic and non-enzymatic sources of ROS generation, as well as a robust antioxidant capacity, to efficiently maintain cellular function and integrity [43]. RBCs endogenously produce ROS via slow autoxidation of Hb to produce methaemoglobin and superoxide, which is subsequently converted by catalase and peroxidases into hydrogen peroxide (H_2_O_2_) and H_2_O. Superoxide and NO rapidly react to form peroxynitrite (ONOO^-^), which in turn may oxidise proteins, lipids and DNA and impact vascular signalling pathways [43]. Several NOX isoforms have been detected in human RBCs, including NOX1, 2, 4 and 5 [24,44,45]. In our model, our findings point to NOX4 as a source of elevated ROS in the vascular microenvironment, such that RBC-derived NO bioactivity prevents elevations in RBC NOX4-derived ROS.

Arginase 1 has been found to be expressed in both human and rodent RBCs, the activity of which influences NO bioactivity, and nitrate and nitrite release [22]. Many studies have highlighted altered RBC arginase homeostasis in cardiometabolic pathologies [41]. Arginase competes with NOS enzymes for their common substrate, l-Arginine. Therefore, we investigated the potential role of dysregulated arginase contributing to the observed RBC eNOS KO-induced endothelial dysfunction. Indeed, following pharmacological arginase inhibition, we observed significant improvement in endothelial function comparable to control vessels. Similarly, co-incubation of eNOS KO RBCs with endothelial-specific Arg1 KO vessels prevented the observed endothelial dysfunction. However, RBC arginase activity was comparable between WT and eNOS KO RBCs. This suggests that the interaction of eNOS KO RBCs and the endothelium is dependent on vascular arginase.

Previous studies have evidenced a interaction between ROS and vascular arginase I, whereby ROS [46], including H_2_O_2_ [47] and ONOO^-^ [48,49], can induce up-regulation of vascular arginase activity and expression. More recently, in RBCs from T2D patients this mechanism has been evidenced, whereby T2D RBCs induced elevated vascular arginase activity and expression in a ROS-dependent manner, which contributed to endothelial dysfunction [24,25]. Considering the observed role of RBC NOX4-derived ROS in development of a vascular arginase-dependent endothelial dysfunction, we can speculate that in the present model vascular arginase is upregulated via ROS, stimulated by a lack of RBC-derived NO bioactivity.

In contrast to our findings, aortas from RBC-specific eNOS KO mice [21] or chimeras with deficient RBC eNOS [20], did not exhibit endothelial dysfunction *ex vivo*. However, RBC eNOS was evidenced to influence plasma NO metabolites, and play a role in vascular function at the level of resistance arteries, highlighting the role of RBC eNOS *in vivo*. One could argue that the latter could be a result of increased contact between RBCs and the endothelium in resistance vs conduit arteries, which facilitates efficient RBC-endothelial communication *in vivo.* Similarly, our *ex vivo* model facilitated this close interaction between RBCs and the endothelium of the aorta, mimicking the intimacy between RBC and endothelium within resistance arteries. Indeed, when RBCs from eNOS KO mice and aortae from WT mice were separated during the co-incubation period, endothelial function remained intact. The 18 h timepoint utilised in the present study, captures the acute impact of erythrocrine function in vascular homeostasis, which could be of particular clinical relevance in acute cardiovascular events. One can also speculate upon potential compensatory endothelial mechanisms *in vivo* at the level of conduit arteries, which, following longer-term exposure to eNOS KO RBCs, may result in a reduction in their deleterious communication. Indeed, Zotti et al. [50] provided evidence supporting the endothelium as the predominant source of erythrocyte HbNO utilising an EPR approach. In our model, RBCs from global eNOS KO mice were utilised, which, due to their sole exposure to an eNOS KO endothelium, may retain their deleterious capacity. Whereas, in the aforementioned RBC-specific eNOS KO mice [21] and chimeras with deficient RBC eNOS [20], the lack of endothelial dysfunction *ex vivo* at the level of conduit vessels may be a result of the exposure of their RBCs to a healthy endothelium *in vivo*. The protective effect of deta-NONOate, observed in this study, suggests that NO-bioactivity derived from the extra-erythrocytic source may neutralize the detrimental potential of RBCs lacking eNOS. Further studies are needed to investigate this interaction.

Finally, our experimental studies warrant further clinical studies to investigate the potential role and regulation of RBCs in the development and progression of cardiovascular disease. Interestingly, we showed that isolated RBC from patients with PE, similar to eNOS KO mice, induced endothelial dysfunction *ex vivo*, which was not observed when using RBC from women with gestational hypertension. suggesting that this is not linked to elevated blood pressure *per se*. Interestingly, the CP:TF ratio was significantly higher in PE vs both GH and healthy pregnant controls. Similar change in ceroplasmin and transferrin levels have previously been linked to oxidative stress and decreased NO bioavailability [38,40], and associated with cardiovascular disease risk in clinical studies [39].

*In conclusion*, we provide further evidence of the impact of erythrocrine signalling on vascular endothelial homeostasis, whereby RBCs from mice lacking eNOS *per se* and from patients with PE, induce endothelial dysfunction *ex vivo*. In mice, at least, this occurs in part via arginase- and oxidative stress-dependent mechanisms, which disrupts the balance between ROS and NO within the vascular microenvironment. This provides crucial insight into the functional relationship between RBCs and adjacent vessels in health and in cardiovascular disease.

### 4.1 Study limitations

Although translational interpretation of our findings utilising an *ex vivo* model is limited by a lack of flow which is present *in vivo*, a strength of the isolated *ex vivo* model utilised in the present study is the exclusive investigation of erythrocrine function, and the direct impact of RBC eNOS on the function of adjacent vessels. Moreover, in our study RBCs from global eNOS KO mice were utilised, which due to their exposure to an eNOS KO endothelium may influence their phenotype.

### 4. 2 Perspectives

RBC eNOS plays a protective role in the maintenance of endothelial function. These findings provide novel insights into the functional relationship between RBCs and the vasculature in cardiovascular disease, which could potentially be targeted in patients to improve endothelial function. Strategies to prevent propagation of the deleterious crosstalk between RBCs and the endothelium could include boosting of NO bioavailability within the vascular microenvironment, for example via dietary nitrates, to reduce endothelial oxidative stress and restore the protective antioxidant capacity of RBC-derived NO. We believe that the findings of this experimental study motivate and stimulate new clinical studies to investigate the potential role and regulation of RBCs in the development and progression of cardiovascular disease and associated complications.

## Sources of funding

This work was supported by grants from the 10.13039/501100004359Swedish Research Council (2016–01381, 2020-01645), the Swedish Heart and Lung Foundation (20210431, 20170124, 20180568), NovoNordisk (2019#0055026), the 10.13039/501100001648EFSD/Lilly European Diabetes Research Programme (2018#97012), Stockholm County Council Research Funding (ALF, 20190314 & 2022-2023), the 10.13039/501100001659German Research Council Foundation (DFG CO 1305/2-1 to M.M.C-K.), and the Swedish Ekhagastiftelsen (Ekhaga foundation). M.M.C.-K. is a visiting Professor and Wenner-Gren research fellow in the Department of Physiology and Pharmacology, 10.13039/501100004047Karolinska Institutet. Additionally, by research Funds (2–560/2015) and KID funding (2–3707/2013 and 2–1930/2016) from the 10.13039/501100004047Karolinska Institutet, Stockholm, Sweden.

## Declaration of competing interest

The authors declare that they have no known competing financial interests or personal relationships that could have appeared to influence the work reported in this paper.

## Data Availability

Data will be made available on request.
