# Supplementary material for: Red blood cells from endothelial nitric oxide synthase-deficient mice induce vascular dysfunction involving oxidative stress and endothelial arginase I
Source: Redox Biol. 2023 Jan 13;60:102612. doi: 10.1016/j.redox.2023.102612 (PMC9868875; doi:10.1016/j.redox.2023.102612)
Supplement: Multimedia component 1 [file mmc1.docx]

**Supplementary Information for**:

**Red Blood Cells from Endothelial Nitric Oxide Synthase-Deficient Mice Induce Vascular Dysfunction Involving Oxidative Stress and Endothelial Arginase I**

Zhengbing Zhuge, PhD^a,^*, Sarah. McCann Haworth, PhD^a,^*, Carina Nihlén, MSci^a^, Lucas Rannier R.A. Carvalho, PhD^a^, Sophia K. Heuser, PhD^b^, Andrei L. Kleschyov, MD, PhD^a^, Josefine Nasiell, MD, PhD,^c,d^ Miriam M. Cortese-Krott, PhD^a,b^, Eddie Weitzberg, MD, PhD^a,e^, Jon O. Lundberg, MD, PhD^a^, Mattias Carlström, Pharm.D., Ph.D.^a,#^

a. Department of Physiology and Pharmacology, Karolinska Institutet, Stockholm, Sweden

b. Myocardial Infarction Research Laboratory, Division of Cardiology, Pulmonology and Vascular Medicine, Medical Faculty, Heinrich-Heine-University, Düsseldorf, Germany.

c. Department of Clinical Sciences, Karolinska Institutet, Stockholm, Sweden

d. Department of Obstetrics and Gynecology, Danderyd Hospital, Stockholm, Sweden

e. Department of Perioperative Medicine and Intensive Care, Karolinska University Hospital, Stockholm, Sweden

* Equal contribution; ^#^ Corresponding author

**This file includes:**

- Supplementary Methods

- Supplementary Figures (S1 to S9) & Table (S1)

- Supplementary Figure & Table Legends

- References for SI reference citations

**Correspondence**:

Mattias Carlström (Pharm.D., Ph.D., Professor)

Karolinska Institutet, Biomedicum, 5B, Dept. of Physiology & Pharmacology

Solnavägen 9, S-171 77, Stockholm, Sweden

Phone: +46-790 680782

E-mail: [mattias.carlstrom@ki.se](mailto:mattias.carlstrom@ki.se)

***Supplementary Methods***

*1. EPR spin trapping of vascular NO*

Spin trapping of vascular NO via colloid Fe(II)-DETC in freshly isolated aortic rings from WT and eNOS KO mice was conducted via EPR as previously described [1]. Briefly, aortic rings were incubated in a 24-well plate with colloid Fe(II)-DETC; A23187, diluted to a final concentration of 10 µM in Krebs buffer, at 37°C for 1 hour. EPR spectroscopy was performed on a table-top ESR spectrometer, Magnettech-Bruker Miniscope MS5000. Recordings were made at 77K using a Dewar flask (Wilmad, USA). Instrument settings were microwave power: 10mW; amplitude modulation: 1mT; modulation frequency: 100kHz; sweep time: 60s; number of scans: 4.

*2. Arginase activity in RBCs*

Arginase activity was determined via its ability to convert L-Arginine to Urea. Ghost cells from washed murine RBCs were prepared using an ice-cold hypotonic buffer (PBS diluted 1:27 in distilled water) to induce opening of pores in the RBC membranes. The RBC ghosts were then lysed using RIPA buffer supplemented with protease inhibitors (11836170001; Roche). 50 µl, corresponding to 100 ug protein, of the lysed ghost cells were added to 75 µl of Tris-HCl (50 mM, pH 7.5) containing 10 mM MnCl_2_. The mixture was activated by heating for 10 min at 56°C. Then, each sample was incubated at 37°C for 3 h with L-Arginine (50ul of 500 mM Tris HCl at pH 9.7). The reaction was stopped by adding 400 µl of an acid solution (H_2_SO_4_–H_3_PO_4_–H_2_O = 1∶3:7). 25 ul of α-isonitrosopropiophenone (9% in ethanol) was added to each sample and standard and the mixture was heated at 100°C for 60 min. The urea concentration was determined at 550 nm using spectrophotometry and calculated as Urea nmol/mg protein/min.

*3. Effects of a supernatant-derived factor on vascular function*

To investigate the potential of a mediating factor in the observed endothelial phenotype, washed RBCs from WT and eNOS KO mice were incubated overnight with DMEM media (17.5 mM Glucose) in a cell culture incubator (37°C with 5% CO_2_). Subsequently, samples were centrifuged (5 mins x 900 g) and supernatants were co-incubated with WT aortic rings overnight, to mimic the aforementioned *ex vivo* preparations. Following incubation, the WT aortic rings were washed and mounted onto the pins of the myograph system and vascular reactivity was assessed (*described above*).

*4. Role of haemolysis on vascular function*

To assess the potential for elevated haemolysis accounting for the observed eNOS KO RBC-derived endothelial dysfunction phenotype, washed RBCs from both WT and eNOS KO mice were completely lysed (using 1:50 dilution with distilled H_2_0), and further diluted to 0, 0.005, 0.015, 0.05, 0.15, 0.5 and 1.5% lysed RBCs in DMEM media (17.5 mM Glucose). Different dilutions of lysed RBC samples were then co-incubated overnight with WT aortic rings, and subsequently, vascular reactivity was assessed via myography (*described above*).

*5. Quantification of supernatant haemoglobin*

Supernatants were collected for haemoglobin quantification. In addition, supernatants post-coincubation of Control (DMEM 17.5 mM), WT RBCs (10%) and eNOS RBCs (10%) with WT aorta were collected for haemoglobin quantification. The Drabkin method was utilised for colorimetric quantification of total haemoglobin in supernatant samples, utilising thew reagent kit according to manufacturer’s instructions (D5641; Sigma, USA). Briefly, 20ul of supernatant samples were added to 500ul of Drabkin’s solution (1:25 ratio) were added to a 96-well plate, briefly mixed, and incubated at room temperature for 15 mins to allow the reaction to stabilize. Conversion of haemoglobin to cyanmethemoglobin was quantified via absorbance at 540nM, measured spectrophotometrically utilising a microplate reader (SpectraMAX iD3; Molecular devices, CA, USA). Absorbance (540nM) values were interpolated into a cyanmethemoglobin standard curve (0-120 mg/mL) to determine sample cyanmethemoglobin concentration (mg/mL).

***Supplementary Figures***

**Figure S1.**

***
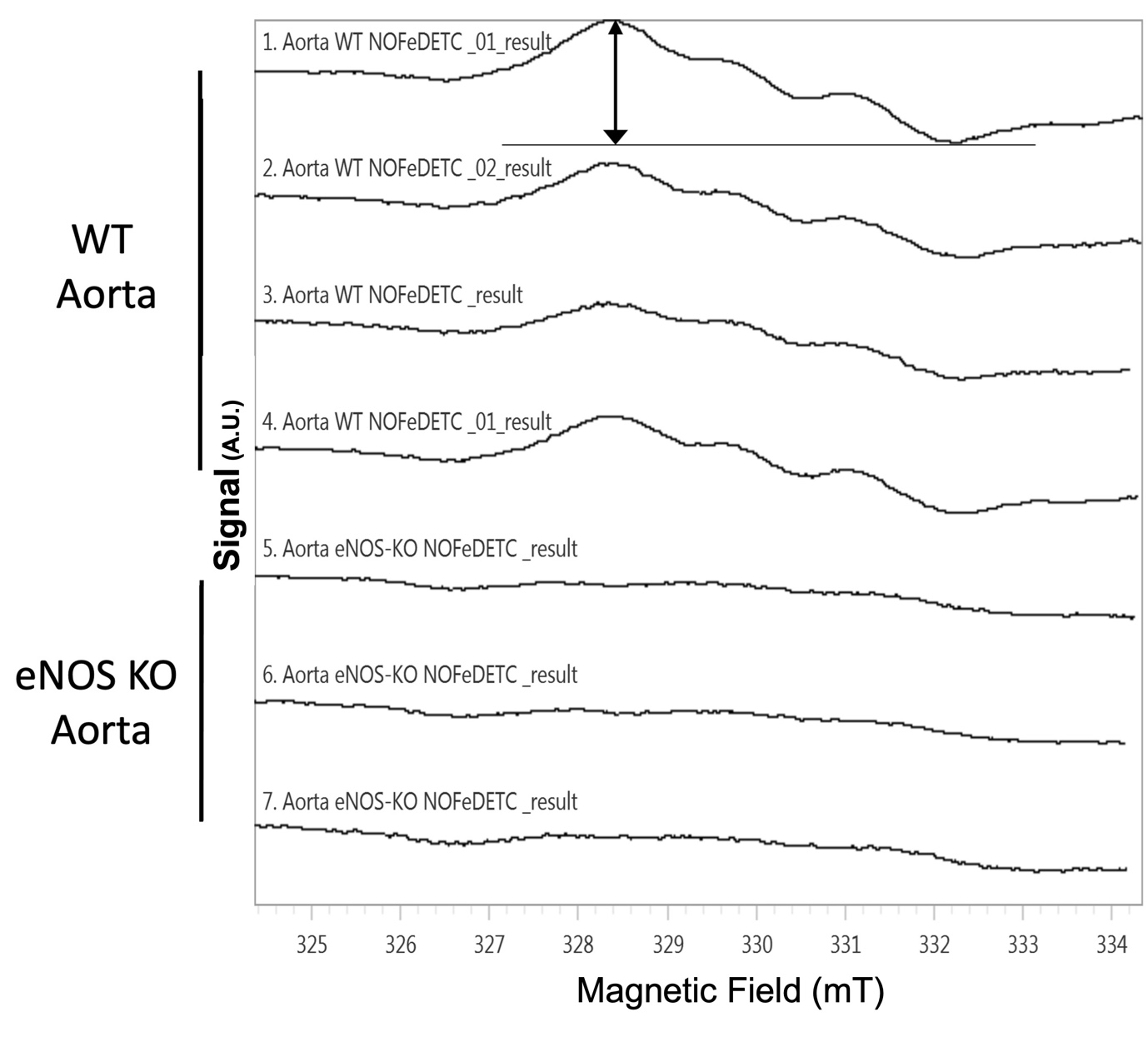
***

**Figure S1. Vascular NO production was not detected in aortas from Global eNOS KO mice.** Characteristic EPR spectra of NO-Fe(II)-DETC2 accumulated in mouse aorta after incubation (37°C; 1hr) with A23187 (10 µM) and colloid Fe(II)-DETC (100 µM). Spectra were recorded at 77K. Instrument settings indicated under supplemental methods.

**Figure S2.**

**Figure S2. RBCs from DelCre global eNOS KO mice induce endothelial dysfunction. (A)** Endothelial-dependent vasorelaxation (% of Phenylephrine plateau; PE) response curve to acetylcholine (Ach; log mol/L) and **(B)** endothelial-independent vasorelaxation (% of phenylephrine plateau; PE) response curve to nitroprusside (SNP) of mouse aortic rings following overnight incubation with Control, RBC from an WT mouse (eNOS^flox/flox^; WT RBC) or RBC from a DelCre eNOS KO mouse (eNOS KO RBC). Control and eNOS KO aorta incubation denotes incubation of a WT or eNOS KO aorta, respectively, with DMEM media without RBC. Data expressed as mean ± SEM; Control, n=4; WT aorta + WT RBC, n=5; WT aorta + eNOS KO RBC, n=5; eNOS KO aorta; n=3, analysed via ordinary 2-way ANOVA with multiple comparisons and Tukey’s post-hoc test. Statistical significance defined as **p*<0.05, ***p*<0.01; Comparisons of WT aorta + eNOS KO RBC *vs Control; # vs WT aorta + WT RBC; † vs eNOS KO aorta.

**Figure S3.**

**Figure S3. RBCs from female eNOS KO mice induce endothelial dysfunction. (A)** Endothelial-dependent vasorelaxation (% of Phenylephrine plateau; PE) response curve to acetylcholine (Ach; log mol/L) and **(B)** endothelial-independent vasorelaxation (% of phenylephrine plateau; PE) response curve to nitroprusside (SNP) of female mouse aortic rings following overnight incubation with Control, RBC from a female WT mouse (WT RBC) or RBC from a female eNOS KO mouse (eNOS KO RBC). Control and eNOS KO aorta incubation denotes incubation of a female WT or eNOS KO aorta, respectively, with DMEM media without RBC. Data expressed as mean ± SEM; Control, n=7; WT aorta + WT RBC, n=11; WT aorta + eNOS KO RBC, n=18; eNOS KO aorta; n=10, analysed via ordinary 2-way ANOVA with multiple comparisons and Tukey’s post-hoc test. Statistical significance defined as **p*<0.05, ***p*<0.01; Comparisons of WT aorta + eNOS KO RBC *vs Control; # vs WT aorta + WT RBC; † vs eNOS KO aorta.

**Figure S4.**

**Figure S4. iNOS not involved in eNOS KO RBC-induced endothelial dysfunction. (A, C)** Endothelial-dependent vasorelaxation (% of Phenylephrine plateau; PE) response curve to acetylcholine (Ach; log mol/L) and **(B, D)** endothelial-independent vasorelaxation (% of phenylephrine plateau; PE) response curve to nitroprusside (SNP) of WT mouse aortic rings following overnight incubation with Control, **(A, B)** RBC from an eNOS KO mouse (eNOS KO RBC) **(C, D)** RBC from a WT mouse (WT RBC) +/- specific iNOS inhibitor (0.1uM; 1400W). Control incubation denotes incubation with DMEM media without RBC. Data expressed as mean ± SEM; n=3-6 per group; analysed via ordinary 2-way ANOVA with multiple comparisons and Dunnetts post-hoc test. Statistical significance defined as, **p*<0.05, ***p*<0.01; ****p*<0.005; comparisons of Control vs *WT aorta + eNOS KO RBC; # vs WT aorta + eNOS KO RBC + 1400W.

**Figure S5.**

**Figure S5. Vascular iNOS not involved in eNOS KO RBC-induced endothelial dysfunction. (A, C)** Endothelial-dependent vasorelaxation (% of Phenylephrine plateau; PE) response curve to acetylcholine (ACh; log mol/L) and **(B, D)** endothelial-independent vasorelaxation (% of phenylephrine plateau) response curve to nitroprusside (SNP) of WT mouse aortic rings following overnight incubation with Control, **(A, B)** RBC from an eNOS KO mouse (eNOS KO RBC) **(C, D)** RBC from a WT mouse (WT RBC) +/- specific iNOS inhibitor (0.1uM; 1400W) added acutely to the myograph chambers and incubated for 30 mins prior to beginning the myography protocol. Control incubation denotes incubation with DMEM media without RBC. Data expressed as mean ± SEM; n=3-6 per group; analysed via ordinary 2-way ANOVA with multiple comparisons and Dunnetts post-hoc test. Statistical significance defined as, **p*<0.05, ***p*<0.01; ****p*<0.005; comparisons of Control vs *WT aorta + eNOS KO RBC; # vs WT aorta + eNOS KO RBC + 1400W.

**Figure S6.**

**Figure S6. Arginase activity in membrane fractions of red blood cells (RBC) from wild-type (WT) and eNOS KO mice.** Data expressed as mean±SEM; WT (n=15), eNOS KO (n=10). Analysed with Student’s t test for unpaired observations. Statistical significance defined as *p*<0.05.

**Figure S7.**

**Figure S7. Close eNOS KO RBC-vascular proximity required for induction of endothelial dysfunction. (A, C)** Endothelial- dependent vasorelaxation (% of Phenylephrine plateau; PE) response curve to acetylcholine and **(B, D)** endothelial independent vasorelaxation (% of PE plateau) to sodium nitroprusside (SNP) of mouse aortic rings following **(A, B)** overnight (~18hrs) incubation in transwell (0.4 μm) permeable inserts with Control, WT RBC and eNOS KO RBC; and **(C, D)** following overnight incubation with supernatant’s collected following 18 hours incubation with Control, WT RBCs supernatant or eNOS KO RBCs supernatant. Data expressed as mean ± SEM; **(A, B)** n=3, **(C, D)** n=4 per group; analysed via Two- way ANOVA with Tukey post-test. Statistical significance defined as **p*<0.05.

**Figure S8.**

**Figure S8. Elevated haemolysis of eNOS KO RBCs unlikely to induce endothelial dysfunction.** Endothelial-dependent vasorelaxation in response to acetylcholine (ACh) of WT aortic rings following overnight (18hr) co-incubation with lysed **(A)** WT RBCs and **(B)** eNOS KO RBCs, at increasing concentrations (%). 0% haematocrit denotes incubation with DMEM media without RBC. % denotes % lysed RBCs in DMEM media. **(C)** Cyanmethemoglobin (mg/ml) concentrations of RBC lysates co-incubated with WT aortae. **(D)** Cyanmethemoglobin (mg/ml) concentrations of supernatants collected post-coincubation with WT aortae and Control, WT RBC (10% haematocrit) or eNOS KO RBC (10% haematocrit). Data expressed as mean ± SEM; (A, B, C, D) n=3; (E) Control, n=6; WT RBC, n=9; eNOS KO RBC, n=17. Statistical significance defined as **p*<0.05, ***p*<0.01; *****p*<0.001, (A, B) vs 0% RBC lysates.

**Figure S9.**


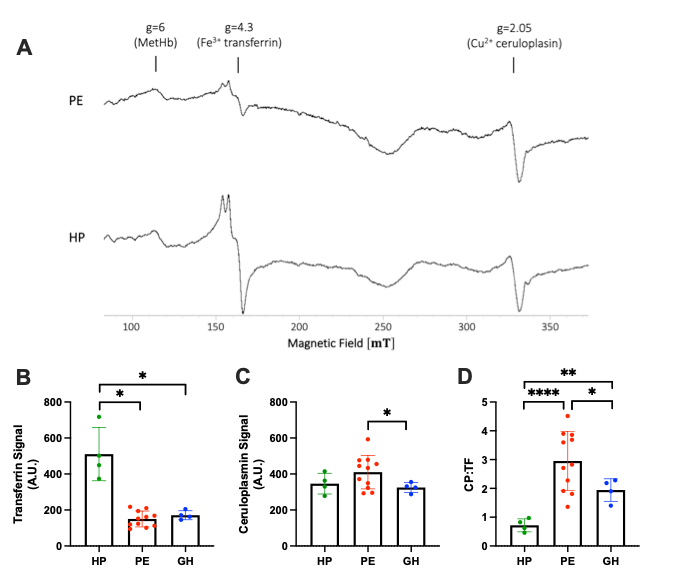


**Figure S9. Assessment of Fe^3+^-Transferrin and Cu^2+^-Ceruloplasmin EPR signals in whole blood. (A)** Representative EPR spectra for met-Hb, Fe^3+^-transferrin and Cu^2+^-ceruloplasmin in whole blood from patient with preeclampsia (PE) and healthy pregnant (HP) women. Whole blood samples from HP, PE and gestationally hypertensive (GH) women were quantified for **(B)** Fe^3+^-Transferrin (TF), and **(C)** Cu^2+^-Ceroplasmin (CP), and the **(D)** calculated ratio between CP and TF. Data analysed with 2-way ANOVA with Bonferroni *post-hoc* testing. HP; *n*=4, PE; *n*=11, GH; *n*=4 per group. Statistical significance defined as **p*<0.05; *****p*<0.00001.

**Table S1. Clinical characteristics of RBC donors**

|  | **HP** | **GH** | **PE** | ***P* Value***** |
| --- | --- | --- | --- | --- |
|  | **(*n*=5)** | **(*n*=3)** | **(*n*=19)** |  |
| **Maternal Age** *Mean (SD)* | 33 ± 3 | 36 ± 8 | 33 ± 5 | 0.5099 |
| **Gestational age at delivery** (*wk)* | 40 (2) | 40 (4) | 37 (3)* | 0.0108^#^ |
| **Body Mass Index** (kg/m^2^) *Mean (SD)* | 21.0 ± 2.4 | 25.1 ± 4.4 | 27.8 ± 6.8 | 0.0841 |
| **Office SBP,** (mmHg) *Mean (SD) [C.I]* | 111 ± 11 | 134 ± 1 | 141 ± 15*** | 0.0009^###^ |
| **Office DBP,** (mmHg) *Mean (SD) [C.I]* | 68 ± 5 | 92 ± 5** | 88 ± 9**** | <0.0001^####^ |

HP, healthy pregnant; GH, gestational hypertension; PE, preeclampsia; BMI, body mass index; *n*, number of participants; wk, week; CI, 95% confidence intervals; SD, standard deviation. Parametric variables: Chi-squared test (dichotomous variables); 1-way ANOVA (continuous data); Nonparametric variables; Kruskal-Wallis test (continuous data). Statistical significance defined as **p*<0.05, ***p*<0.01; *****p*<0.001; vs HP, ﻿and # between all three groups in row.

***Supplimentary References***

[1] A.L. Kleschyov, H. Mollnau, M. Oelze, T. Meinertz, Y. Huang, D.G. Harrison, T. Munzel, Spin trapping of vascular nitric oxide using colloid Fe(II)-diethyldithiocarbamate, Biochem Biophys Res Commun 275(2) (2000) 672-7.
